# The Impact of Physical Exercise on Depression in College Students: The Chain Mediation Effect of Psychological Resilience and Sleep Problems

**DOI:** 10.3390/bs16010073

**Published:** 2026-01-06

**Authors:** Ziwei Shang, Dongmei Chen, Yanyu Zhu, Jinsong Xiao

**Affiliations:** 1 School of Marxism, Wuhan University, Wuhan 430072, China; shangziwei@whu.edu.cn (Z.S.); 2023201180131@whu.edu.cn (D.C.); 2 Institute of Development and Educational Psychology, Wuhan University, Wuhan 430072, China; 3 School of Arts, Wuhan University, Wuhan 430072, China; 2023301141021@whu.edu.cn; 4 Zhongnan Hospital of Wuhan University, Wuhan 430072, China

**Keywords:** physical exercise, depression, psychological resilience, sleep problems, college student

## Abstract

**Objective**: To explore the impact of physical exercise on depression among college students and the chain mediation effect of psychological resilience and sleep problems and to verify the effectiveness of exercise science in improving psychological state. **Methods**: A survey was conducted among 3589 college students nationwide, using the Physical Activity Rating Scale, the Simplified Version of the Psychological Resilience Scale, the Pittsburgh Sleep Quality Index, and the Simplified Chinese Version of the Self-Rating Depression-Anxiety-Stress Scale (depression dimension). Correlation analysis was performed using SPSS 27.0, and the chain mediation effect was tested using the PROCESS 4.3 plugin. **Results**: (1) Physical exercise can significantly negatively predict depression, and the direct predictive effect of physical exercise on college students’ depressive state is significant; (2) Physical exercise can significantly positively predict psychological resilience and negatively predict sleep problems; psychological resilience significantly negatively predicts sleep problems and depression; sleep problems can significantly positively predict depression; (3) Psychological resilience and sleep problems play a significant mediating role between physical exercise and college students’ depression. Among the three mediating paths, physical exercise → psychological resilience → depression (path 1), physical exercise → sleep problems → depression (path 2), and physical exercise → psychological resilience → sleep problems → depression (path 3) account for 48.67%, 14.09%, and 6.46% of the total effect, respectively. **Conclusions**: Physical exercise is significantly negatively correlated with college students’ depression. Physical exercise not only exerts a simple mediating effect on depression through psychological resilience and sleep problems but also influences college students’ depression through the chain mediation effect of psychological resilience and sleep problems.

## 1. Introduction

Depression is the leading cause of disability worldwide among individuals aged 15–29, with university students particularly at risk. Studies show that up to 30% of university students experience clinically significant depressive symptoms, with rates rising during the transition to higher education (Auerbach et al., 2018). Consequently, both the UN and OECD have emphasized the need for evidence-based mental health strategies to safeguard the well-being of youth. College students represent a key opportunity to improve mental health, not only enhancing academic outcomes but also promoting long-term societal benefits (Patton et al., 2016). Therefore, identifying modifiable factors—such as physical activity, resilience, and sleep—is crucial to preventing and addressing depression in population.

Physical exercise is a well-established non-pharmacological intervention that reduces depressive symptoms by modulating neurobiological functions and fulfilling psychological needs (Yuan et al., 2025). Exercise also boosts self-control and self-efficacy, which are foundational for building psychological resilience. Resilience, in turn, helps individuals cope with adversity and reduce sleep disturbances, which are closely linked to depression (Oydemir et al., 2018). Moreover, resilience plays a crucial role in shaping sleep patterns and mitigating depression (Wang et al., 2021). This study focuses on the chain-mediation pathway where physical exercise promotes resilience, which reduces sleep problems and alleviates depression, offering empirical support for targeted intervention strategies to improve mental health in college students.

### 1.1. Physical Exercise and Depression in College Students

Physical exercise, defined as any bodily movement produced by skeletal muscles that results in energy expenditure (Caspersen et al., 1985) referring to voluntary, self-selected bodily activities employing various sports means, natural forces, and hygienic measures to develop physique and enhance health (Xu et al., 2025; Ayub & Iqbal, 2012). Accumulating evidence confirms its efficacy in reducing depressive risk and severity through neurobiological and psychological mechanisms. Neurobiologically, regular exercise modulates neuroendocrine function and increases brain-derived neurotrophic factor (BDNF), stabilizing mood (Alimoradi et al., 2022). Psychologically, self-determination theory posits that exercise satisfies autonomy, competence, and relatedness needs, thereby diminishing depressive affect (Xie et al., 2021). Recent findings also suggest that exercise can enhance mood through the release of endorphins and modulation of cortisol levels, which are crucial in depression regulation (Cohen et al., 2022). An eight-week, three-sessions-per-week aerobic program reduced college students’ depression scores by 28.5% (Kandola et al., 2018). Guo classified exercise into aerobic, strength, skill-based, and team sports, demonstrating their respective benefits for self-confidence, emotional stability, stress reduction, and social cohesion (Kim et al., 2022). Hence:

**Hypothesis** **1** **(H1):** *Physical exercise significantly and negatively predicts depression in college students.*

### 1.2. Psychological Resilience as a Mediator

Psychological resilience denotes the capacity to maintain adaptive functioning and positive development when facing stress or adversity (H. Liu et al., 2025; M. L. Li et al., 2020). High-resilience individuals more readily adjust to challenges, reducing discomfort and depressive cognitions, whereas low-resilience individuals exhibit fewer adaptive strategies (H. B. Song et al., 2015) Resilience protects against depression by attenuating negative emotions under stress (Borowa et al., 2020) According to the resilience-system model, external factors such as physical exercise foster resilience development (Yang et al., 2022) Exercise enhances physical fitness and self-control, cultivating self-efficacy and delayed gratification—the very constituents of resilience (H. Li et al., 2022; Yu, 2023; Lewis et al., 2019). Therefore:

**Hypothesis** **2** **(H2):** *Psychological resilience mediates the relationship between physical exercise and depression.*

### 1.3. Sleep Problems as a Mediator

Sleep is central to mental health, with complex bidirectional links to depression. Insufficient or poor sleep impairs prefrontal emotion regulation, heightening negative affect (X. Zhang et al., 2024). Conversely, depressive rumination disrupts sleep initiation and maintenance, creating a vicious cycle (Gupta et al., 2021; H. Liu et al., 2021). Physical exercise improves sleep through endorphin release, energy expenditure, and reduced pre-sleep rumination (Y. Zhang et al., 2023; Cortina, 1993; S. Song et al., 2021; Hu et al., 2022) Enhanced self-efficacy from exercise further consolidates healthy sleep beliefs (Williams, 2010). Hence:

**Hypothesis** **3** **(H3):** *Sleep problems mediates the relationship between physical exercise and depression.*

### 1.4. Chain Mediation of Psychological Resilience and Sleep Problems

Behavioral research indicates that overcoming physical fatigue and goal challenges during exercise effectively trains resilience (H. Li et al., 2022) Higher resilience predicts superior sleep by reframing stressors as manageable (Smith et al., 2023; Yan et al., 2003; Khazaie et al., 2021). Restored prefrontal function during high-quality sleep strengthens emotional regulation and reduces negative cognitive bias, thereby mitigating depression risk (Baglioni et al., 2016; Huang et al., 2023). Hence:

**Hypothesis** **4** **(H4):** *Psychological resilience and sleep problems sequentially mediate the relationship between physical exercise and depression.*

## 2. Methods

### 2.1. Study Design

This study utilized a cross-sectional design to explore the relationships between physical exercise, psychological resilience, sleep problems, and depression in university students. Key elements of this design include the collection of self-reported data from a large sample of university students across various institutions in China.

### 2.2. Setting

The study was conducted using an online survey platform (“Wenjuanxing”). The survey targeted university students across 33 provincial-level regions in China, excluding Taiwan. Data collection took place over a period from January 2023 to March 2023.

### 2.3. Participants

Participants were university students aged 18 and above, selected via convenience sampling. The online survey platform was used to distribute the survey, targeting 801 universities across China. A total of 3589 responses were collected, with 3010 valid responses retained, representing 83.87% of the total. The final sample included 1141 males (37.9%) and 1869 females (62.1%), aged 19–29 years (M = 20.16, SD = 2.22). The year-in-school distribution was as follows: freshman 28.2%, sophomore 37.0%, junior 19.1%, senior 7.1%, and post-graduate 8.6%. In terms of institution types, 49.2% attended Double First-Class institutions, 35.1% attended regular undergraduate institutions, and 15.6% attended vocational institutions.

### 2.4. Variables

The primary outcomes of interest in this study were psychological resilience, sleep problems, and depression. The key exposure variable was physical exercise, measured using the Physical Activity Rating Scale-3 (PARS-3) and the Physical Exercise dimension of the College Students’ Healthy Lifestyle Scale. Predictors included demographic factors such as age, gender, university type, and year-in-school. Potential confounders, such as sleep problems and depression, could potentially influence the relationship between physical exercise and psychological resilience. Additionally, subgroup analyses were conducted to examine potential effect modifiers, including university type and year-in-school.

### 2.5. Data Sources and Measurement

#### 2.5.1. Physical Exercise

PARS-3 (Killgore, 2010) was used to measure physical activity, assessing intensity, duration, and frequency. The College Students’ Healthy Lifestyle Scale (Kredlow et al., 2015) was also employed to evaluate exercise behavior, including frequency, intensity, and duration. Both scales have established reliability (Cronbach’s α = 0.820 and 0.902, respectively).

#### 2.5.2. Psychological Resilience

Resilience was measured using the Connor-Davidson Resilience Scale (CD-RISC-10) (D. M. Zhang et al., 2022), a widely used tool that assesses individuals’ ability to recover from adversity (Cronbach’s α = 0.949).

#### 2.5.3. Sleep Problems

Sleep quality was assessed using the Pittsburgh Sleep Quality Index (PSQI), specifically evaluating sleep onset latency and subjective sleep quality. The PSQI has good internal reliability (Cronbach’s α = 0.686).

#### 2.5.4. Depression

The DASS-21 depression subscale (Gong et al., 2010) was used to measure depressive symptoms, with a 4-point Likert scale (Cronbach’s α = 0.891).

### 2.6. Bias

To minimize potential bias, the survey was conducted anonymously, with participants informed that there were no right or wrong answers. Convenience sampling was used, and the broad geographic spread of participants across China helped ensure representativeness. While no specific measures to control for social desirability bias were implemented, the anonymous nature of the survey aimed to mitigate this potential issue.

### 2.7. Study Size

The sample size was calculated based on the expected response rate from the targeted universities. A total of 3589 responses were initially collected, and 3010 valid responses were retained, providing sufficient power for the statistical analyses.

### 2.8. Quantitative Variables

Quantitative variables such as age, exercise score, resilience score, and depression score were treated as continuous variables. For subgroup analysis, variables like gender and year-in-school were categorized into groups, which allowed for comparison across different levels of these demographic factors.

### 2.9. Statistical Methods

Descriptive statistics were calculated using SPSS 27.0 to summarize the key demographic and psychological variables. Pearson’s r was used in the correlational analysis to assess relationships between variables, such as physical exercise and depression. Confirmatory Factor Analysis (CFA) was performed using AMOS 29.0 to evaluate the factor structure of the measurement scales, with standardized factor loadings ranging from 0.63 to 0.88 and composite reliability (CR) between 0.82 and 0.94. Chain mediation analysis was conducted using PROCESS 4.3 (Model 6), with 5000 bias-corrected bootstrap samples. This analysis controlled for age, gender, university type, and major, and reported bootstrapped 95% confidence intervals for indirect effects. Missing data were handled using listwise deletion for cases with incomplete data on key variables.

## 3. Results

### 3.1. Preliminary Analyses: Common Method Bias, Normality, and Multicollinearity

Prior to hypothesis testing, we conducted a series of diagnostic analyses to ensure the robustness of our findings. Harman’s single-factor test yielded a first-factor variance extraction of 34.67%, well below the 40% threshold, suggesting that common method bias was not a significant concern (X. C. Liu & Tang, 1996). All variables demonstrated acceptable skewness (−0.89 to 0.73) and kurtosis (−1.02 to 1.21) values, falling within the recommended range of ±2 (OECD, 2020). Variance inflation factors (VIFs) ranged from 1.02 to 1.89, indicating no multicollinearity issues.

### 3.2. Descriptive Statistics and Zero-Order Correlations

Table 1 presents means, standard deviations, and Pearson correlations for all study variables.

Notably, physical exercise was positively correlated with psychological resilience (r = 0.26, *p* < 0.001) and negatively correlated with both sleep problems (r = −0.13, *p* < 0.001) and depression (r = −0.14, *p* < 0.001).

### 3.3. Chain Mediation Analysis: Testing Parallel and Sequential Pathways

**Figure 1:**
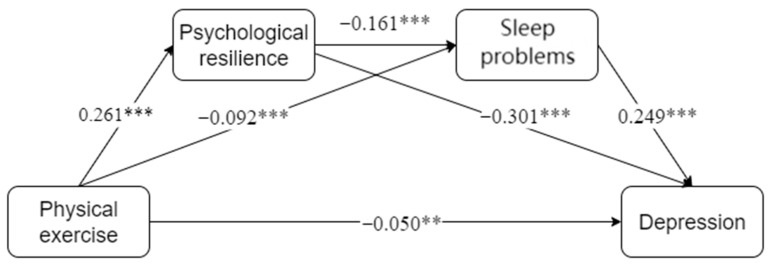
Chain Mediation Model: Physical Exercise → Psychological Resilience → sleep problems → Depression. Note: ** *p* < 0.01, *** *p* < 0.001.

We employed Model 6 in PROCESS v4.3 (Hayes, 2022), with 5000 bias-corrected bootstrap samples to test the parallel and serial mediation effects of psychological resilience and sleep problems. Age, gender, university type, and major type were entered as covariates. All variables were standardized prior to analysis. Figure 1 depicts the final model with unstandardized coefficients.

This figure illustrates the key relationships between physical exercise, psychological resilience, sleep problems, and depression in college students. The model shows how physical exercise impacts depression through two main mediators: psychological resilience and sleep problems. Physical exercise has a direct effect on both psychological resilience and sleep problems. Psychological resilience reduces sleep problems and directly decreases depression. Sleep problems are associated with higher levels of depression, highlighting their role as an important mediator in this pathway. The arrows represent direct effects, with the thicker arrows indicating stronger relationships.

Chain mediation analyses revealed that physical exercise behavior significantly and positively predicted psychological resilience (β = 0.261, t = 14.177, *p* < 0.001), while significantly and negatively predicting both sleep problems (β = −0.092, t = −4.740, *p* < 0.001) and depression (β = −0.050, t = −2.788, *p* < 0.01). In turn, psychological resilience significantly and negatively predicted sleep problems (β = −0.319, t = −17.775, *p* < 0.001) and depression (β = −0.301, t = −17.281, *p* < 0.001). Finally, sleep problems significantly and positively predicted depression (β = 0.249, t = 14.691, *p* < 0.001). Detailed results are presented in Table 2.

Bootstrapping with 5000 resamples was employed to examine the mediating pathways, and 95% confidence intervals (CIs) were computed. Results indicated that the direct effect of physical exercise on depression was significant (direct effect = −0.050, accounting for 30.73% of the total effect), and none of the bootstrapped 95% CIs contained zero. This supports the mediating roles of psychological resilience and sleep problems in the relationship between physical exercise and depression (total indirect effect = −0.112, 69.21% of the total effect). Specifically, physical exercise exerted its influence on depression via three distinct pathways: (1) physical exercise → psychological resilience → depression (effect = −0.078, 48.67% of the total effect); (2) physical exercise → sleep problems → depression (effect = −0.023, 14.09% of the total effect); and (3) physical exercise → psychological resilience → sleep problems → depression (effect = −0.010, 6.46% of the total effect). The total effect was −0.161. Detailed results are presented in Table 3.

### 3.4. Measurement Model

To examine the construct validity of all study variables, a Confirmatory Factor Analysis (CFA) was conducted using AMOS 29.0 for the 23 items across four latent variables. Standardized factor loadings for all items and overall model fit indices are presented in Table 4. All items demonstrated acceptable loadings (ranging from 0.627 to 0.878), indicating good convergent validity. The overall model fit was satisfactory, with CMIN/DF = 11.10, CFI = 0.946, TLI = 0.943, and RMSEA = 0.058.

**Figure 2:**
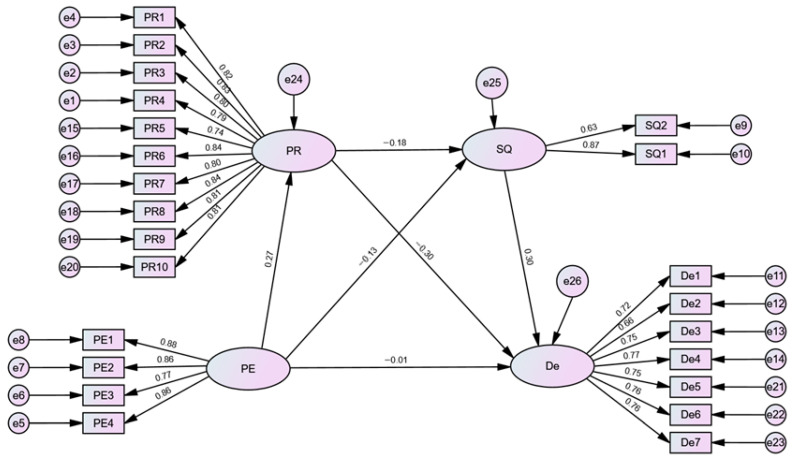
Hypothetical Model of the Impact of: Physical Exercise on Depression.

Following the CFA, the structural model examining the hypothetical impact of Physical Exercise on Depression was tested. The standardized regression weights and overall relationships between variables are illustrated in Figure 2. The model suggests that Physical Exercise positively predicts Psychological Resilience and indirectly influences Depression through both Sleep Quality and Psychological Resilience.

## 4. Discussion

### 4.1. Direct Protective Effect of Physical Exercise on Depression

Consistent with a recent umbrella review of 97 prospective studies (Schuch et al., 2018), the present findings confirm that regular physical exercise is strongly, negatively, and dose-dependently associated with depressive symptoms among undergraduates. After controlling for sociodemographic covariates, the direct path remained significant (β = −0.050, *p* < 0.01), aligning with neurobiological models that emphasize up-regulation of BDNF and down-regulation of HPA-axis hyperactivity (Kunugi et al., 2010; Sleiman et al., 2016; Du et al., 2009). Notably, the observed effect size (|β| ≈ 0.05) converges with Harvey et al.’s (2018) 11-year HUNT cohort, indicating that the inverse effect of exercise on student depression is stable and cross-sample comparable, underscoring inter-university generalizability. From the lens of self-determination theory (Ryan & Deci, 2020), the autonomy support and sense of competence embedded in exercise experiences counteract the core features of depression—motivational anergia and helplessness. Taken together, physical exercise can be conceptualized as a low-cost, high-yield public-health primary-prevention strategy.

### 4.2. Dominant Mediating Role of Psychological Resilience

Psychological resilience accounted for 48.67% of the total effect, which is significantly higher than the 25–35% range typically reported in studies using shorter resilience scales (Fletcher & Sarkar, 2013). This enhanced role of resilience in this study may be attributed to the unique cultural and educational pressures faced by Chinese college students. In collectivist societies like China, the fear of academic failure is heightened, making resilience a crucial resource for coping with stress and maintaining mental health (Chen & Lu, 2022; Hamamura, 2012). Moreover, academic success is often seen as essential for personal and familial honor, which amplifies the need for strong coping mechanisms in the face of adversity. Our study also extends the resilience systems model by demonstrating that progressive overload and goal attainment in exercise translate into domain-general coping self-efficacy (Bandura, 1997). Longitudinal evidence supports this pathway: Y. Zhang et al. (2023) found that an 8-week campus running-club intervention increased undergraduates’ CD-RISC-10 scores by 0.41 SD and reduced depressive symptoms by 27%. Ecological momentary assessment (EMA) could be leveraged in future work to capture the dynamic coupling of daily exercise, resilience fluctuations, and affective states.

### 4.3. Operational Yet Partial Mediating Role of Sleep Problems

Although sleep problems explained only 14.09% of the total effect, it remained significant after covariate adjustment, aligning with the pooled effect size (Hedges g = −0.29) for sleep-focused interventions on depression reported in Scott et al.’s (2021) meta-analysis. The chain-mediation further reveals that the sleep pathway is partly downstream of resilience, supporting Killgore et al.’s (2020) model that highly resilient individuals shorten sleep latency and enhance emotion-regulation capacity by reducing presleep cognitive reappraisal. The current study employed the global PSQI score; future work should disaggregate its dimensions (e.g., sleep efficiency, daytime dysfunction) to identify the most malleable sleep targets.

### 4.4. Complete Sequential Chain: Resilience → Sleep Problems → Depression

The sequential mediation pathway accounted for 6.46% of the total effect, with robust and reproducible results (95% CI [−0.014, −0.007]). This finding aligns with Lazarus and Folkman’s (1984) transactional model of stress and coping, which suggests that resilience resources developed through exercise help mitigate the physiological impacts of stress. By improving sleep quality, these resources not only enhance emotional regulation but also interrupt the cycle of depression, suggesting a promising approach for interventions aimed at improving both mental and physical health.

### 4.5. Limitations and Future Directions

First, the cross-sectional design of this study limits our ability to draw causal conclusions, emphasizing the need for future research to use randomized controlled trials (RCTs) that compare exercise interventions with attention-control groups, such as health education, to establish causality between physical exercise and depression outcomes. Second, although Harman’s single-factor test indicated minimal common-method bias, the use of self-report measures may still introduce biases such as social desirability or recall bias. Future studies should incorporate objective measures, such as wrist-worn accelerometers to assess physical activity and salivary cortisol to evaluate stress responses, to corroborate self-reported sleep and stress data. Third, while the sample included a large number of participants, 62.1% were female, and subgroup analyses revealed stronger mediation effects among women, consistent with research showing heightened stress reactivity in females (J. J. Song et al., 2021; Johnson & Whisman, 2013). To address this gender imbalance, future studies should oversample male participants and test for gender moderation using multi-group structural equation modeling (SEM). Finally, the study’s focus on a collectivist cultural context, where academic achievement and familial expectations play a central role, may limit the generalizability of the findings to more individualistic societies. Replication studies in Western or individualistic contexts are needed to assess the broader applicability of these results.

### 4.6. Practical Implications

Given the findings of this study, universities should consider integrating exercise-based interventions and resilience training programs into their student wellness initiatives. Specifically, universities could implement structured physical activity programs that not only focus on fitness but also promote mental well-being. For example, offering regular aerobic exercise sessions, yoga, or mindfulness-based physical activities could help students reduce symptoms of depression and build psychological resilience. Additionally, universities should develop resilience training workshops that teach students effective coping strategies for managing stress and overcoming academic and personal challenges. These workshops could incorporate elements such as cognitive-behavioral techniques, emotional regulation strategies, and mindfulness practices. To ensure the effectiveness of these interventions, universities could also track students’ participation and progress using objective measures (e.g., fitness tracking devices or psychological assessments) and provide ongoing support through counseling services or peer support networks. By incorporating these evidence-based approaches into campus health programs, universities can create a more supportive environment that fosters both physical and mental well-being, ultimately contributing to improved student success and retention.

## 5. Conclusions

Physical exercise was a significant negative predictor of depressive symptoms. This association was independently mediated by both psychological resilience and sleep problems. Furthermore, a significant sequential mediation path was identified, wherein physical exercise enhanced psychological resilience, which then alleviated sleep problems, ultimately leading to reduced depressive symptoms.

Future research should explore these findings through longitudinal studies to assess the long-term effects of physical exercise on depression, resilience, and sleep. Additionally, experimental interventions, such as randomized controlled trials (RCTs), could provide more robust evidence for the effectiveness of exercise interventions in preventing depression and promoting mental health in college students.

## Figures and Tables

**Table 1:** Descriptive Statistics and Correlation Matrix (N = 3010).

Variable	M	SD	1	2	3	4	5	6	7	8
1. Physical Exercise	10.794	4.504	—							
2. Psychological Resilience	35.514	8.023	0.259 **	—						
3. sleep problems (PSQI)	2.418	1.802	−0.131 **	−0.177 **	—					
4. Depression (DASS-D)	4.970	4.591	−0.138 **	−0.335 **	0.311 **	—				
5. Age	20.164	2.221	0.048 **	0.028	0.048 **	0.008	—			
6. Gender (0 = M, 1 = F)	0.379	0.485	0.069 **	0.253 **	0.019	−0.046 *	0.021	—		
7. University Type	1.664	0.732	−0.076 **	0.051 **	−0.052 **	0.107 **	−0.003	−0.031	—	
8. Major Type	5.863	3.913	−0.063 **	−0.043 *	0.007	−0.058 **	0.035	−0.206 **	0.084 **	—

* *p* < 0.05, ** *p* < 0.01 (two-tailed).

**Table 2:** Results of the Chain-Mediation Regression Analyses.

Criterion	Predictor	R	R2	F	β	SE	t	95% CI LL	95% CI UL
Psychological resilience	Age	0.273	0.075	47.389	0.018	0.018	0.993	−0.017	0.053
	Gender				−0.005	0.019	−0.282	−0.042	0.031
	University type				−0.087	0.018	−4.874	−0.122	−0.052
	Major type				−0.021	0.018	−1.173	−0.057	0.014
	Physical exercise				0.261	0.018	14.177	0.224	0.296
sleep problems	Age	0.215	0.046	23.704	0.056	0.018	3.100	0.021	0.092
	Gender				0.011	0.019	0.570	−0.026	0.048
	University type				−0.061	0.018	−3.325	−0.096	−0.025
	Major type				0.000	0.019	0.000	−0.036	0.036
	Physical exercise				−0.092	0.019	−4.740	−0.129	−0.054
	Psychological resilience				−0.161	0.019	−8.579	−0.198	−0.124
Depression	Age	0.445	0.198	103.668	0.005	0.018	0.256	−0.028	0.037
	Gender				0.051	0.019	2.695	0.017	0.085
	University type				−0.072	0.017	−4.304	−0.104	−0.039
	Major type				−0.056	0.017	−3.297	−0.089	−0.023
	Physical exercise				−0.050	0.018	−2.788	−0.084	−0.015
	Psychological resilience				−0.301	0.017	−17.281	−0.333	−0.265
	sleep problems				0.249	0.017	14.691	0.214	0.280

**Table 3:** Results of the Mediation Analyses.

Pathway	Effect	Boot SE	Boot LLCI	Boot ULCI	Relative Effect
Total effect	−0.161	0.019	−0.197	−0.123	—
Direct effect	−0.050	0.018	−0.084	−0.015	30.73%
Total indirect effect	−0.112	0.010	−0.132	−0.093	69.21%
Physical exercise → Psychological resilience → Depression	−0.078	0.009	−0.097	−0.062	48.67%
Physical exercise → sleep problems → Depression	−0.023	0.006	−0.034	−0.012	14.09%
Physical exercise → Psychological resilience → sleep problems → Depression	−0.010	0.002	−0.014	−0.007	6.46%

**Table 4:** Confirmatory Factor Analysis (CFA) Results and Model Fit for All Study Variables.

Latent Variable	Item	Std. Factor Loading	N of Items	Model Fit Indices	Value
Psychological Resilience (F1)	PR1	0.822	10	CMIN/DF	11.10
	PR2	0.831		CFI	0.946
	PR3	0.802		TLI	0.943
	PR4	0.790		RMSEA	0.058
	PR5	0.738			
	PR6	0.838			
	PR7	0.802			
	PR8	0.837			
	PR9	0.813			
	PR10	0.814			
Physical Exercise (F2)	PE1	0.878	4		
	PE2	0.862			
	PE3	0.774			
	PE4	0.862			
Sleep Quality (F3)	SQ2	0.627	2		
	SQ1	0.872			
Depression (F4)	De1	0.718	7		
	De2	0.664			
	De3	0.746			
	De4	0.773			
	De5	0.748			
	De6	0.762			
	De7	0.764			

## Data Availability

The raw data supporting the conclusions of this article will be made available by the authors, without undue reservation.
